# Thor: a platform for cell-level investigation of spatial transcriptomics and histology

**DOI:** 10.21203/rs.3.rs-4909620/v1

**Published:** 2025-03-10

**Authors:** Pengzhi Zhang, Weiqing Chen, Tu Nhi Tran, Minghao Zhou, Kaylee N. Carter, Ibrahem Kandel, Shengyu Li, Xen Ping Hoi, Keith Youker, Li Lai, Qianqian Song, Yu Yang, Fotis Nikolos, Keith Syson Chan, Guangyu Wang

**Affiliations:** 1. Center for Bioinformatics and Computational Biology, Houston Methodist Research Institute, Houston, TX, 77030, USA; 2. Center for Cardiovascular Regeneration, Houston Methodist Research Institute, Houston, TX, 77030, USA; 3. Center for RNA Therapeutics, Houston Methodist Research Institute, Houston, TX, 77030, USA; 4. Department of Cardiothoracic Surgery, Weill Cornell Medicine, Cornell University, New York, NY, 10065, USA; 5. Department of Physiology, Biophysics & Systems Biology, Weill Cornell Graduate School of Medical Science, Weill Cornell Medicine, Cornell University, New York, NY, 10065, USA; 6. Department of Health Outcomes and Biomedical Informatics, University of Florida, Gainesville, FL, 32610, USA; 7. Department of Urology, Houston Methodist Research Institute, Houston, TX, 77030, USA; 8. Spatial Omics Core, Neal Cancer Center, Houston Methodist Research Institute, Houston, TX, 77030, USA; 9. Graduate Program in Biomedical Sciences, Cedars-Sinai Medical Center, Los Angeles, CA 90069, USA; 10. Department of Cardiovascular Sciences, Houston Methodist Research Institute, Houston, TX, 77030, USA; 11. Department of Pathology, Immunology and Laboratory Medicine, College of Medicine, University of Florida, Gainesville, FL, 32608, USA

## Abstract

Spatial transcriptomics integrates transcriptomics data with histological tissue images, offering deeper insights into cellular organization and molecular functions. However, existing computational platforms mainly focus on genomic analysis, leaving a gap in the seamless integration of genomic and image analysis. To address this, we introduce Thor, a comprehensive computational platform for multi-modal analysis of spatial transcriptomics and histological images. Thor utilizes an anti-shrinking Markov diffusion method to infer single-cell spatial transcriptomes from spot-level data, effectively integrating cell morphology with spatial transcriptomics. The platform features 10 modules designed for cell-level genomic and image analysis. Additionally, we present Mjolnir, a web-based tool for interactive tissue analysis using vivid gigapixel images that display information on histology, gene expression, pathway enrichment, and immune response. Thor’s accuracy was validated through simulations and ISH, MERFISH, Xenium, and Stereo-seq datasets. To demonstrate its versatility, we applied Thor for joint genomic-histology analysis across various datasets. In in-house heart failure patient samples, Thor identified a regenerative signature in heart failure, with protein presence confirmed in blood vessels through immunofluorescence staining. Thor also revealed the layered structure of the mouse olfactory bulb, performed unbiased screening of breast cancer hallmarks, elucidated the heterogeneity of immune responses, and annotated fibrotic regions in multiple heart failure zones using a semi-supervised approach. Furthermore, Thor imputed high-resolution spatial transcriptomics data in an in-house bladder cancer sample sequenced using Visium HD, uncovering stronger spatial patterns that align more closely with histology. Bridging the gap between genomic and image analysis in spatial biology, Thor offers a powerful tool for comprehensive cellular and molecular analysis.

## Introduction

The complex organization of cells within tissues is profoundly connected to their biological function. This underpins the widespread utility of histological images in health and disease. The development of computational methods empowered by deep learning on histological images has drastically enhanced efficiency and accuracy in tissue analysis in diverse applications^1^, including automated cancer diagnosis^2^, survival prediction^3^, histopathology image classification and retrieval^4^, tissue segmentation^5, 6^, nucleus and cell segmentation^7–9^, and *in silico* staining^10^. Furthermore, rapid advancements in high-throughput technologies such as RNA sequencing (RNA-seq) and whole genome sequencing (WGS) are transforming the landscape of conventional histological analysis, offering unprecedented insights beyond tissue images. For example, recent research has demonstrated that the integration of histological images with genomic biomarker mutations and biological pathways leads to accurate predictions of survival across diverse conditions^3, 11^. In the evolving landscape of biological investigation, spatially resolved molecular technologies have become a pivotal focus for unraveling cellular diversity, tissue organization, and functions. Spatial omics data have been incorporated and routinely acquired by programs such as the human cell atlas (HCA) and the human biomolecular atlas program (HuBMAP), advancing the construction of comprehensive spatial maps featuring various biomolecules, including RNA, proteins, and metabolites^12, 13^. A widely adopted molecular technology is spatial transcriptomics (ST), which involves slicing tissues into thin layers for hematoxylin and eosin (H&E) staining and spatial sequencing, enabling simultaneous investigation of tissue/cellular phenotype and molecular mechanism on the same slide.

Recent efforts to advance ST analysis have focused on incorporating spatial neighborhood information^14^, or integrating histology images^15–17^. However, these tools typically operate at subspot or superpixel spatial scales, which do not correspond to individual cells, hindering biologically relevant insights – particularly in contexts requiring cell-level data, such as analyzing ligand-receptor interactions. Another branch of ST analysis frameworks addresses cellular heterogeneity by resolving cell-type compositions within spatial spots^18–20^. However, these approaches do not infer cell-level gene expression and are further restricted by the quality and availability of scRNA-seq reference data, especially for formalin-fixed paraffin-embedded (FFPE) tissues where transcriptomic data quality is often compromised. While emerging methods enable cellular-level histological structure analysis^21, 22^, similarly they do not generate single-cell resolution gene expression matrices, thereby excluding them from downstream functional or molecular analyses. Moreover, those platforms are mostly tailored to specific tasks (e.g. deconvolution), whereas comprehensive analysis platforms (e.g. Seurat) prioritize -omics analysis without deeply analyzing histopathological images^23–25^.

To meet the urgent need for jointly analyzing genomics and histology, we present a multi-modal platform – Thor – for bridging and exploring cellular phenotypes and molecular insights. Thor enhances the incorporation of morphology and transcriptome data of individual cells by inferring cell-resolution transcriptome from spot-level ST data using an anti-shrinking Markov graph diffusion method. Moreover, Thor features extensible modules for comprehensive genomic analyses, such as immune response, functional pathway enrichment, transcription factor (TF) activity, and copy number variation (CNV), alongside tissue analyses such as semi-supervised tissue annotation and nucleus detection. Additionally, we develop Mjolnir, a user-friendly web-based platform for interactive exploration of cellular organization and pathogenesis in tissues, on a laptop, with no coding required.

We elucidated the principles of Thor and rigorously assessed its effectiveness and accuracy through simulations and various datasets, obtained from high-resolution experimental methods, including in situ hybridization (ISH), multiplexed error-robust fluorescence in situ hybridization (MERFISH)^26^, spatio-temporal enhanced resolution omics-sequencing (Stereo-seq)^27^, and Xenium^28^. Thor outperformed state-of-the-art methods in predicting cell-level ST on a breast cancer dataset using Xenium data as the ground truth. We analyzed a mouse olfactory bulb (MOB) tissue, human breast cancer tissues, and multi-sample heart failure patient tissues. Thor revealed a refined layered structure in MOB and identified distinct gene modules. In heart failure, Thor quantified fibrotic regions across different heart zones. Furthermore, we collected in-house heart failure samples from patients who received a left ventricular assist device (LVAD) implantation to study the signature genes in vascular regeneration. We identified regenerative signatures in heart failure and validated them through immunofluorescence (IF) staining. In breast cancer, Thor conducted an unbiased screening of breast cancer hallmarks, uncovering the intricate heterogeneity of immune responses in tumor regions. In summary, Thor enables comprehensive interpretation of ST data at the single-cell and whole-transcriptome levels, delivering advanced functional insights, and providing an interactive interface for in-depth analyses.

## Results

### Thor infers cell-resolution spatial transcriptome for multi-modal analysis

**Figure 1:**
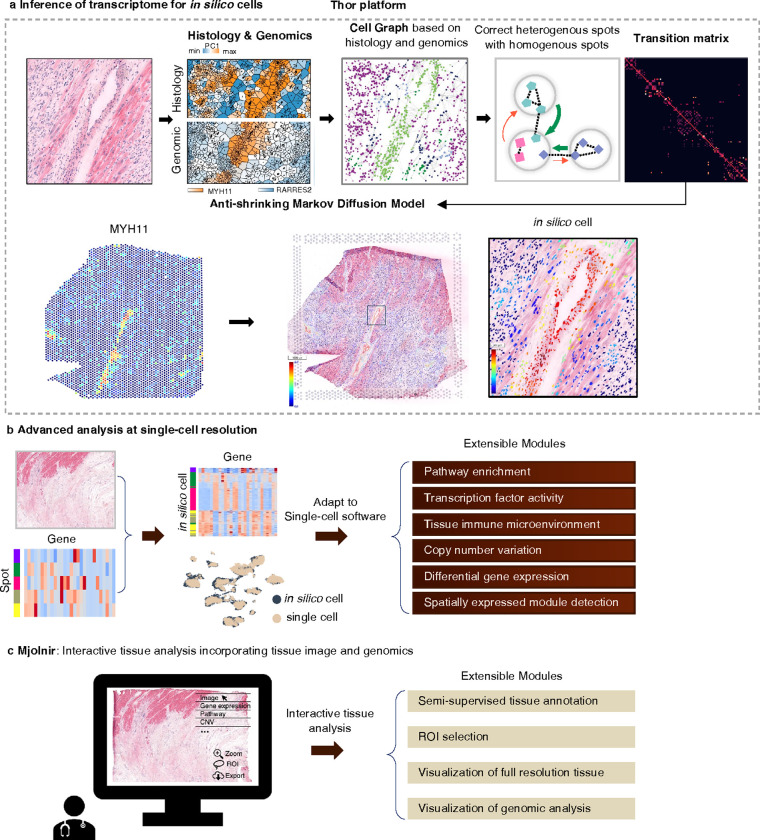
Thor - a software suite for integrated analyses of histology and transcriptomics data at the *in silico* cell level. (a) Histological images and high-throughput sequencing data capture inherent cellular structures at different resolutions and share complementary information. The projection of the histological features to the first principal component highlights the tissue sections at cell resolution; meanwhile, expression patterns of marker genes of the cardiac smooth muscle cells (*MYH11*) and the fibroblast cells (*RARRES2*) demonstrate consistent patterns at spot resolution. The cell-cell network is constructed according to the distances in the combinatory feature space of histology (including location) and transcriptomics. In the example and illustration of cell-cell network, the nodes represent cells, edges represent connections, and the colors indicate cell types. Thor infers single-cell spatial transcriptome by utilizing an anti-shrinking Markov graph diffusion model*.* The expression profile of the marker gene *MYH11* in smooth muscle aligns with the texture of the H&E staining image, as visualized by the Mjolnir web platform. (b) Thor adapts and implements a diversity of modules for advanced single-cell analyses around the inferred spatially resolved whole transcriptome of the *in silico* cells. (c) The Mjolnir platform supports interactive multi-modal tissue analysis.

Histological images and high-throughput sequencing data are widely adopted for various applications^2, 29–31^. Despite their significance, these two sources of information are often examined independently with separate tools. Sequencing-based ST and the paired histological whole slide image (WSI) capture inherent cellular structures in the tissue at different resolutions, providing complementary information. For example, in human heart tissues with MI, we observed that the projection of histological features onto principal components segregated tissues at cellular resolution (Figure 1a, Supplementary Note 1). Similarly, spatial patterns can be discerned through marker gene expression at a coarser resolution (spot level). Clustering results of spots by using either source of features were consistent and complementary, as demonstrated in the human MI samples, a human ductal carcinoma in situ (DCIS) sample, and a MOB sample (See details in Supplementary Note 1). Previous studies also indicated that spatial gene expression can be predicted or refined based on histological images^15–17^. Therefore, we hypothesize that it is feasible to recover cell-level resolution transcriptomics data by learning shared patterns from both the histology and the transcriptome.

Multi-modal analysis in Thor involves two key steps. First, elevating spot-resolution ST data to single-cell resolution (Figure 1a). Second, in-depth genomics and tissue image analyses (Figure 1b–c). In the first step, Thor (i) applies deep learning methods to segment cells/nuclei from the WSI, termed *in silico* cells; (ii) extracts morphological and spot-level transcriptomic features into a combinatory feature space to construct a cell-cell network; (iii) creates a Markov transition matrix, representing the probabilities of transitioning from a cell to every other cell in the system in one step; (iv) infers gene expression of the *in silico* cells by data diffusion with the transition matrix (Figure 1a). Thor represents the cellular patterns using a nearest neighbors graph, where cells are connected according to their distances in the combinatory feature space reflecting the physical separation, and the histological and genomic complexity. The Markov transition matrix is constructed such that information from “homogeneous” spots asymmetrically corrects information from “heterogeneous” spots, where heterogeneity of a spot is determined by the enclosed cells in the combinatory feature space (Figure 1a). In the second step, we establish a standardized genomics analysis framework for in-depth research and clinical practice. The genomics analysis encompasses a wide array of insights, including cell type annotation, immune response analysis, biological functional pathway analysis, differential gene expression analysis, spatially expressed module detection, TF activity analysis, and CNV analysis (Figure 1b). Thor also includes tissue image analysis tools including nucleus segmentation, region of interest (ROI) selection, and semi-supervised ROI annotation. To enhance accessibility and usability, we introduce a web-based platform Mjolnir that seamlessly visualizes both histological images and genomic analyses (Figure 1c). Altogether, Thor elevates tissue analysis by integrating image analysis and genomic insights.

### Thor demonstrates accuracy and robustness in simulation data

We systematically evaluated Thor’s accuracy and robustness under realistic experimental conditions. We simulated expression profiles for 1,000 genes in 6,579 cells, whose spatial positions were extracted from a mouse cerebellum tissue as the ground truth^32^; and based on those cells, we created “spots” by aggregating gene expression levels in cells covered by a spot (Figure S1a, see details in Methods: Simulation details). We assessed Thor’s prediction accuracy by computing the normalized root mean squared error (NRMSE) between the predicted and the ground-truth gene expression values (see Methods for details).

We first evaluated Thor’s performance under suboptimal histology imaging conditions. Two primary issues can impact its accuracy: (i) missed detection of cell nuclei, which commonly occurs in out-of-focus or high-density regions, and (ii) erroneous cell-cell connections resulting from poor histological features. Under ideal conditions with neither cell dropouts nor randomized connections, Thor’s predicted gene expression closely matched the ground truth, yielding a median NRMSE of 0.07 (Figures S1a and S2). Introducing random “missouts” of cells (0%−40%) lead to a slight increase in median NRMSE from 0.07 to 0.075 (Figure S1b), while introducing randomized connections in 30% - 40% of cells modestly increased the median NRMSE to 0.08 (Figure S1b). These findings suggest that Thor maintains robust prediction accuracy even in the presence of substantial missing cells and disrupted cell connections.

Next, we examined the spatial resolution, a critical factor in spatial technologies ranging from subcellular scales to ~100 *μm*. Larger spots lead to greater cell heterogeneity within each spot (Figure S3). When we varied the spot diameter from 25 *μm* to 150 *μm*, Thor accurately predicted single-cell gene expression for spots up to ~100 *μm* in diameter, although the median NRMSE increased to 0.08 at 150 *μm*. To further highlight advantages of our algorithm, we compared Thor against three alternative methods: (a) nearest spot method – assigning gene expression based on the nearest spot; (b) k-nearest neighbors (KNN) smoothing method – assigning gene expression by averaging over the nearest twenty cells; and (c) BayesSpace – assigning gene expression based on local spatial neighborhoods of sub-spots. At 25 *μm*, both the nearest spot method and Thor exhibited high accuracy (median NRMSE 0.06). The nearest spot method’s performance declined sharply as spot size increased beyond 25 *μm*, while Thor remained accurate with the spot size up to 100 *μm*. This suggests that Thor’s superior performance is not solely due to incorporating nucleus segmentation. By contrast, both the KNN smoothing method and BayesSpace performed poorly across all spot sizes, with median NRMSE values of approximately 0.2 (Figure S1c). The KNN smoothing method consistently underperformed, underscoring the benefits of Thor’s shared nearest neighbors cell-cell graph and feature-preserving Markov diffusion approach.

To quantitatively evaluate Thor’s performance under increasing spot complexity, we plotted the mean absolute error (MAE) of each cell against the Shannon entropy of cell type proportions. As spot heterogeneity increased, the MAE for the nearest spot method rose sharply; meanwhile, Thor accurately imputed gene expression for both low (Figure S3c, A) and high (Figure S3c, B, C) heterogeneity spots. Although a subset of cells in highly heterogeneous spots showed a slight increase in MAE (Figure S3c, C), Thor’s error remained much lower than that of the nearest spot method.

Finally, to evaluate Thor’s imputation performance under varying dropout levels, an important challenge in high-resolution spatial transcriptomics, we simulated 15 conditions with dropout ratios ranging from 5% to 60% and categorized them into three regimes: low dropout (<15%), moderate dropout (15–40%), and high dropout (>40%). We then measured cluster separations in principal component analysis (PCA) space using silhouette coefficients. As shown in the PCA plots (Figure S1d), introducing dropouts severely diminished cluster separations in the ground truth data, with silhouette coefficients reduced from 0.8 to near 0. In contrast, Thor-imputed data maintained the silhouette coefficient to above 0.7–0.8 in the low-dropout regime, outperforming the KNN smoothing method and BayesSpace. When dropout ratios rose to the moderate regime, where the ground truth data’s silhouette coefficients declined to 0.1–0.4, Thor-imputed data recovered the cluster separation successfully (silhouette coefficients 0.5–0.6). Even under high-dropout conditions (>40%), Thor’s scores remained substantially above those of KNN smoothing and BayesSpace.

Collectively, these analyses highlight Thor’s accuracy and robustness in the presence of suboptimal histology or transcriptomics data, including high proportions of missed cells, disrupted cell connections, varying spot sizes, and substantial technical dropouts.

### Thor infers accurate gene expression at single-cell resolution

Next, we evaluated Thor on a mouse brain receptor map data acquired by MERFISH. The MERFISH data comprised 483 RNA targets from individual cells (Figure S4a). We simulated Visium-like data within the hippocampus region by creating a grid of evenly spaced “ST spots”. The RNA molecule counts in a synthetic spot were aggregated over the cells covered by the “ST spot”. These synthetic spots contained a mixture of cells of different cell types, particularly within the hippocampal subregions CA1/2/3 and the dentate gyrus (DG; Figure S4a). Thor connected cells of the same cell types by proximity in the morphological feature space and the spatial space, as illustrated by the cell-cell network in CA1 and DG (Figure S5a; note the cell type information was not provided to Thor). Thor successfully predicted cell-level gene expression in these heterogeneous regions evidenced by the profiles of selected marker genes (Figures S4b and S5b). For instance, Thor recovered *Adra1d* expression in CA1 and DG, which was missing in the spot-level data and the BayesSpace result. Furthermore, to gain a global view of the similarity between the *in silico* cells and the MERFISH cells, we projected the high-dimensional gene expression matrices to a joint uniform manifold approximation and projection (UMAP) embedding. The *in silico* cells inferred by Thor seamlessly mixed with the MERFISH cells on UMAP, and the distribution of cell type clusters of the *in silico* cells matched the ground-truth cell types (Figure S4c). As a baseline, mixtures of cell types were aggregated in the spot-level data, resulting in a low silhouette coefficient and Calinski-Harabasz index when mapped to the nearest cells. Thor substantially improved the cell type separation, achieving a silhouette score of 0.45 and a high Calinski-Harabasz index of 10,000, and outperformed BayesSpace by a large margin (Figure S4d).

**Figure 2:**
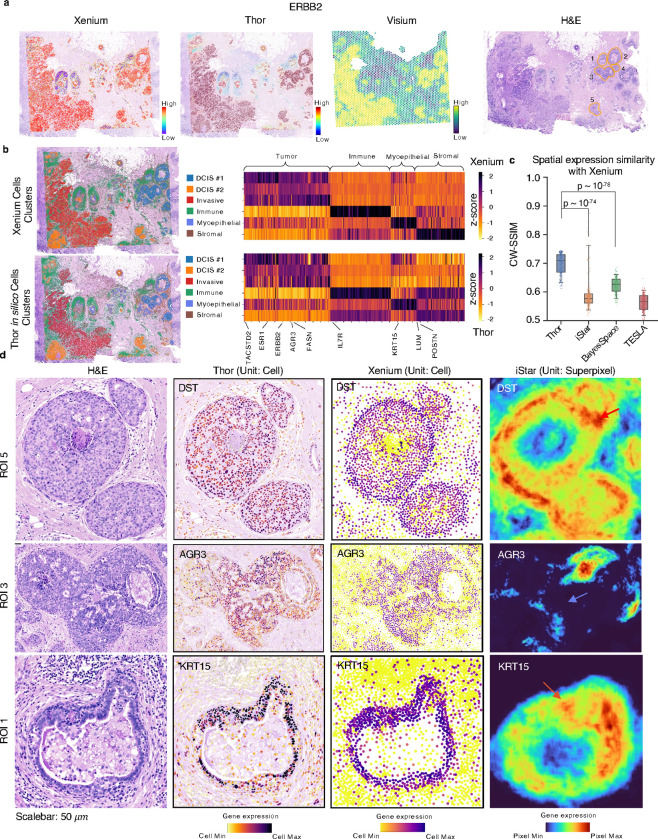
Thor accurately predicts single-cell spatial gene expression in human breast cancer. (a) Spatial gene expression of *in silico* cells inferred from the Visium data and the H&E staining image of a breast cancer tissue by Thor align closely with Xenium data from the adjacent tissue section. The numbers on the H&E staining image mark DCIS regions of interest. (b) Thor-inferred spatial transcriptome of *in silico* cells demonstrate consistent cell clusters with Xenium using scRNA-seq clustering. The cluster annotations were adapted from the original study of the dataset^28^. The mean expression levels of differentially expressed genes in each cluster were visualized using heatmaps. (c) Thor outperforms iStar in the prediction of spatial gene expression. (d) Spatial expression profiles of representative genes at the region of interest level are compared between Thor, iStar, and Xenium. Thor-inferred spatial gene expression closely aligns with the Xenium data, while iStar introduces artifacts at segment boundaries (the red arrows) and in regions with sparse cells (the blue arrow).

We further applied Thor to a Visium dataset of human breast cancer tissue and compared the result against a Xenium reference dataset of the adjacent tissue section^28^. Using transcriptome data from the Visium dataset and the post-Xenium H&E image as input, Thor successfully inferred *in silico* cell-level gene expression. Visually, the spatial patterns of gene expression align closely with Xenium data (Figure 2a). To gain a global view, we clustered the *in silico* cell-level gene expression using conventional single-cell RNA-seq (scRNA-seq) clustering. The same major cell types were identified from the *in silico* cells as from the Xenium data, as evidenced by the spatial distribution of the cell types and the mean expression heatmap of marker genes of each cell type (Figure 2b). Additionally, integrating the predicted *in silico* cells with the Xenium cells showed that cells from the same cell types colocalize from both datasets (Figure S6), indicating Thor’s ability to predict accurate and biologically meaningful cell-level gene expressions.

For a quantitative evaluation, we benchmarked Thor with three state-of-the-art methods of enhancing ST to near-cell resolution^14, 15, 17^. The spatial units vary among those tools (Thor: cell, iStar: superpixel, BayesSpace: subspot, and TESLA: superpixel), therefore, we calculated both image-centric and cell-centric metrics to provide a more complete evaluation. On the one hand, by converting spatial profiles of gene expression data into images, we compared the similarities between the predicted spatial patterns with the Xenium spatial patterns using the metrics structural similarity index measure (SSIM) and root mean squared error (RMSE) of pixel values. On the other hand, by mapping the pixel expression data to the cells using the nearest neighbors approach, we compared the deviations between the resulted cell-level gene expression with the Xenium data using cell-wise RMSE as an additional metric. Thor achieved the highest similarity with the Xenium data on all the metrics (Figures 2c and S7). When using the cell-wise RMSE, the general trend remains, yet the difference between the four methods became less prominent. This is likely because all the gene expression levels including Thor needed to be mapped to the common cell positions (Xenium cells) using nearest neighbors before calculating cell-wise RMSE, which might have smoothed out some intricate details in the spatial pattern, as seen in Figure S7(c-d). Overall, Thor demonstrated significantly better agreement with the Xenium data.

To gain more insights into Thor’s unique advantage, we compared the expression profiles of representative genes with second best performing tool, iStar. Thor and iStar enhanced spatial resolution to (near) cell resolution, iStar at times introduced artifacts, including excessive fusion, for instance, at segment boundaries (Figure 2d, red arrows), and in regions with sparse cells (Figure 2d, blue arrow). For example, the spatial expression of myoepithelial marker *DST* inferred by Thor accurately outlined the boundaries of three DCIS regions in ROI 5 (Figure 2d), as confirmed by the Xenium data and the H&E staining image. While Thor did not maintain the spatial gradient pattern due to misdetection of flat nuclei around certain region boundaries, iStar introduced excessive fusion in the tumor regions, as indicated by the red arrows in Figure 2d. Additional examples are provided in Figures S8–9. These artifacts are likely due to that iStar predicts the expression of super-pixel patches of the whole slide image, rather than a cell. This approach may result in the omission of valuable cellular morphology information. In contrast, Thor takes a fundamentally different approach by considering a cell as the minimum biological unit and can accurately infer single-cell gene expression via a cell-cell network constructed with the integration of transcriptomics and histology data.

### Thor unveils refined tissue structure in mouse olfactory bulb

We extended our evaluation of Thor-inferred gene expression levels on a MOB dataset collected by Visium. We compared the inferred molecular patterns with those acquired from high-resolution techniques, including the ISH images^33^ and Stereo-seq data^27^. Results showed the spatial patterns of gene expression levels inferred by Thor aligned well with both ISH and Stereo-seq data (Figures S10a and S11). For example, *Eomes* is a marker gene of cells in the glomerular layer and mitral layer^34^, as observed in the ISH and Stereo-seq data. However, due to the limited spatial resolution, the spot-level Visium data failed to adequately capture the pattern in the mitral layer and exhibited discontinuities in the glomerular layer. By integrating the high-resolution H&E image with the spot-resolution ST, Thor recovered the spatial patterns marked by *Eomes* in glomerular and mitral layers (Figure S10a). Detailed gene expression profiles from Thor, ISH, Stereo-seq, and Visium, were provided for comparison in Figure S11.

At the whole-transcriptome level, the *in silico* cell clusters dissected six main layers in the MOB, the subependymal zone (SEZ), two granule layers, the mitral layer, the glomerular layer, and the olfactory nerve layer (Figure S10b). We further applied Cell-ID^35^ to infer cell types (see Method; Signature genes are provided in Supplementary Table S1). By integrating ST with spatial locations and histological features, Thor resolved refined neuron subtypes. For example, Thor distinguished granule cells (GCs) between GC-1 and GC-2 subtypes, with GC-1 concentrated in the internal plexiform layer and GC-2 predominantly in the granule cell layer. Additionally, Thor separated mitral cells (M/TCs) into M/TC-1 and M/TC-2 subtypes, with M/TC-2 concentrated in the mitral layer and M/TC-1 extending into the glomerular layer. These results demonstrated Thor’s capability to refine cell type classification by leveraging histology and spatial transcriptomic data.

Leveraging the cell-resolution spatial profiles, we next identified genes with spatially dependent activation patterns and coordinated gene modules using the package Hotspot^36^. The genes in the *in silico* cells formed 8 gene modules reflecting the primary structure of MOB (Figure S10c), with modules ‘2’, ‘4’, ‘7’, and ‘8’ capturing the glomerular layer, the mitral layer, the granule layers, and the olfactory nerve layers, respectively (Figure S10d). Remarkably, module ‘4’ captured the thin mitral layer (thickness < 40 *μm*), indicating successful resolution-enhancement by Thor, enriching a thin layer of the M/TC-2 mitral cell subtype. The gene ontology (GO) pathway enrichment analysis of the layer-specific gene modules suggested a cascade of activities covering odor information sensory, processing, signal transmission, and memory formation in MOB layers. Together, Thor unveiled refined layers in the MOB tissue by accurately inferring cell-level gene expression data, aligning with various experimental measurements.

### Thor supports semi-supervised annotation of fibrotic regions in human myocardial infarction tissues

To better leverage the combinatory space of histological and transcriptomic features, we developed a human-in-the-loop tool for enhanced identification of tissue regions or spatial domains. The semi-supervised annotation tool operates within Mjolnir, enabling researchers to annotate small representative regions using marker gene expression and morphology of cells in gigapixel resolution images. These transcriptome- and morphology-guided annotations can then be quickly propagated across the entire tissue section based on Pearson correlation of the combinatory features, facilitating comprehensive tissue characterization.

We first quantitatively evaluated Thor’s semi-supervised annotation using a cohort of heart tissue samples^37^, which included high-resolution H&E images, high-quality ST data, and spot-level expert annotations for key tissue types in heart including vessel, node, adipose, and fibrosis. Thor’s semi-supervised annotations demonstrated strong concordance with expert annotations, achieving accuracy ranges of 0.94–0.99 for vessels, 0.92–0.98 for nodes, 0.84–0.92 for adipose, and 0.92–0.93 for fibrosis (Figure S12). In contrast, spot-level clustering, even with optimized parameters, struggled to distinguish structures such as vessels (enriched with smooth muscle cells) from certain myocardium regions (Figure S13). These results suggest Thor enhances spatial tissue annotation by integrating histology with transcriptomics, surpassing spot-level clustering.

**Figure 3:**
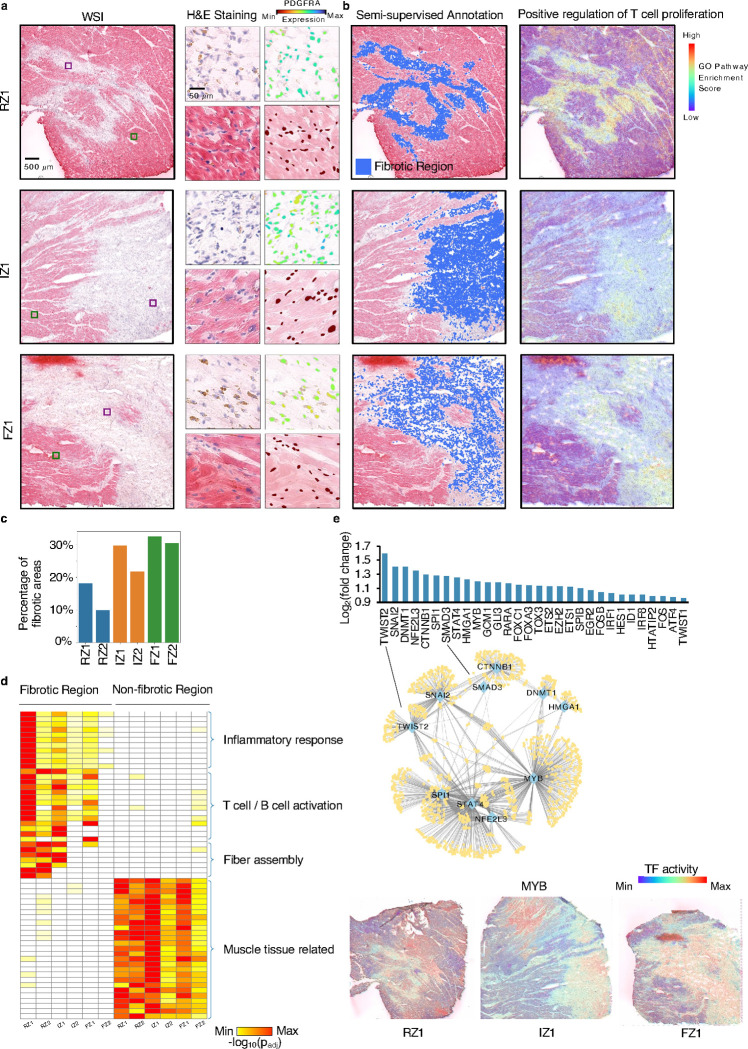
Thor detects fibrotic regions in multiple human heart tissues with MI. (a) H&E staining images of tissues from a remote zone (RZ1), an ischaemic zone (IZ1), and a fibrotic zone (FZ1). Purple and green squares mark curated ROIs and are annotated as fibrotic and non-fibrotic regions. Close-up views of the cell morphology and inferred cellular expression of the fibroblast marker gene PDGFRA are provided for the curated ROIs. (b) Mjolnir-annotated fibrotic regions (blue) are visualized on the H&E staining images. T cell proliferation pathway enrichment scores are calculated based on the top highly expressed genes in each cell. (c) Barplot the percentages of the fibrotic regions in all six samples. (d) Heatmap of the GO pathway enrichment based on the up-regulated DEGs (fold change > 2, adjusted p_value < 0.01 using t-test) in the fibrotic region compared to the non-fibrotic region in each sample, and the up-regulated DEGs (fold change > 2, adjusted p_value < 0.01 using t-test) in the non-fibrotic region compared to the fibrotic region in each sample. (e) TF activity is inferred from the *in silico* cell spatial transcriptome. We use RTN (R package) for the transcriptional network inference and Cytoscape for network visualization.

Next, we applied Thor to analyze six myocardial infarction (MI) patient samples, comprising two necrotic zones (ischemic zone, IZ), two unaffected zones (remote zone, RZ), and two late-stage fibrotic zones (FZ), to enable granular characterization of these distinct tissue zones in heart failure. Using the Mjolnir platform, we first defined an ROI based on fibroblast marker gene expression (*PDGFRA* and *FBLN2*) and morphological patterns in an H&E image. Thor then automatically extended the curated ROIs by identifying similar cells in the entire tissue. The expression profiles of representative genes, including fibroblast marker genes and cardiac muscle-associated genes, displayed coherent patterns in the curated ROIs and the discovered cells (Figures 3a and S14–17). Such semi-supervised annotation revealed dense fibrotic areas and shallow areas which were otherwise difficult to identify manually (Figures 3b, S16). The resulting fractions of fibrotic areas in the six samples increased in the order of RZ, IZ, and FZ (Figure 3c).

The precisely annotated fibrotic regions then enabled unbiased functional analysis. For each sample, we performed differential gene expression analysis between cells in the fibrotic and non-fibrotic regions (lists of differentially expressed genes are provided in Supplementary Table S2), followed by GO pathway enrichment analysis. Irrespective of sample zones, the fibrotic regions showed significant enrichment of pathways such as positive regulation of T cell proliferation, fibroblast proliferation, stress fiber assembly, and collagen fibril organization, whereas myocardium-related pathways were enriched in non-fibrotic regions (Figures 3a, d and S18a). These findings align with previous evidence of T cell proliferation and fibroblast-mediated T cell activation in cardiac settings^38, 39^. Interestingly, the fibrotic regions of RZ samples demonstrated more pronounced inflammation and fibrosis, likely reflecting heterogeneous progression of ischemic injury among the patient samples. After myocardial infarction, tissue in the immediate infarct area often undergoes rapid cell death and necrosis, whereas distant/remote zones may experience a delayed and prolonged inflammatory and fibrotic response^40, 41^. While IZ and FZ contained the largest proportions of fibrotic regions at the whole tissue level (Figure 3c), those findings demonstrate that functionally distinct fibrotic domains can exist outside necrotic regions.

To identify regulatory factors influencing those fibrotic regions, we estimated TF activities by utilizing a gene regulatory network database^42^. Compared to non-fibrotic regions, the most prominently activated TFs induced critical pathways, such as epithelial-mesenchymal transition (*TWIST2* and *SNAI2*) and immune response (*STAT4* and *MYB*; Figures 3e and S18b). The detected top regulating TFs agreed with existing studies: *SMAD3* has been identified as a principal mediator of the fibrotic response to activate cardiac fibroblasts^43^; *SPI1* has been reported as an essential orchestrator of the pro-fibrotic gene expression program in multiple human organs^44^.

Overall, Thor’s semi-supervised annotation provides a more nuanced view by integrating morphological features with transcriptomics. This approach refines fibrotic tissue boundaries, highlights subtle variations in fibrotic progression, and provides functional insights into the molecular drivers of post-infarction cardiac fibrosis.

### Thor discovers regenerative signature in heart failure

Spot-level spatial transcriptomics often struggles to reveal intricate patterns in small or narrow regions due to limitations in spatial resolution. One such example is to identify the regenerative signatures in vessel regions. Thor allows for the exploration of gene expression in cell-resolution spatial contexts by predicting gene expression in cells detected from the histological images, thereby enhancing the ability to uncover intricate patterns.

**Figure 4:**
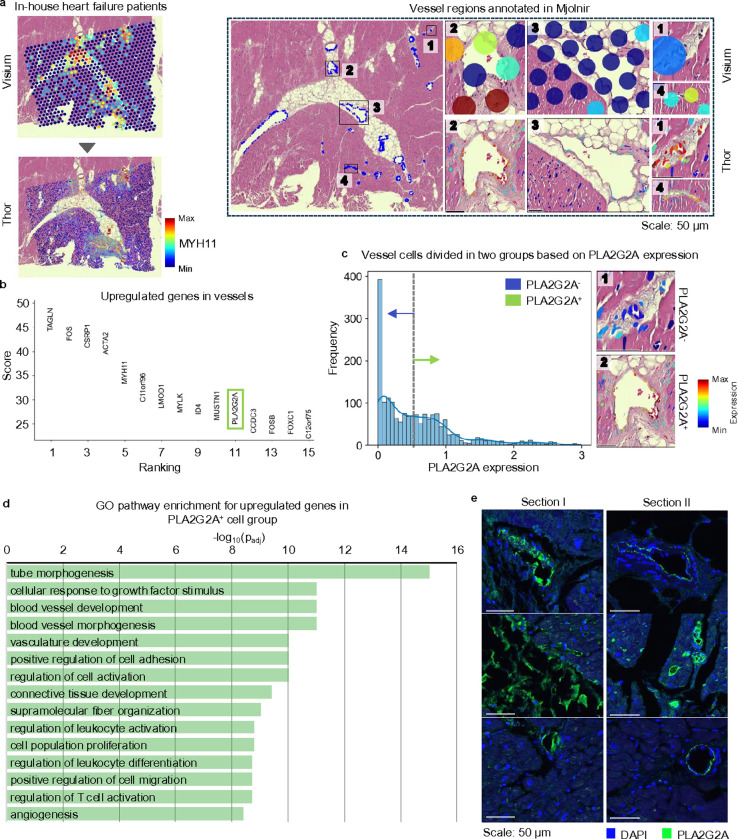
Thor identifies regenerative signatures in vessels in human heart failure. (a) Thor infers cell-level gene expression, expression of the smooth muscle marker *MYH11* are visualized at the spot level and the cell level on sample I tissue. Utilizing Mjolnir, vessel regions are annotated. The expression of *MYH11* in selected vessels (labelled 1–4) is recovered by Thor, where there exhibits low expression of *MYH11* at the spot resolution. (b) The upregulated genes in the vessels shared by two samples are ranked according to the gene scores. (c) Cells in the vessel regions are divided into two groups according to *PLA2G2A* expression levels. A cutoff of 0.5 is used, where the first and the second Gaussian distributions overlap. (d) GO pathway enrichment using the top 500 upregulated DEGs (with the lowest adjusted p_values) in the *PLA2G2A*^*+*^ cells. (e) IF staining views of protein level *PLA2G2A* expression in post-LVAD patient tissues.

In patients with advanced heart failure, LVADs are commonly used before heart transplantation for cardiac support which provide evident improvement in the structure and function of the heart^45, 46^. Thus, we applied Thor to in-house heart tissues collected from post-LVAD implantation patients to identify genes driving regenerative remodeling. As the vasculature system plays an important role in cardiac recovery^47^, we prioritized our analysis in the vascular regions. Blood vessels typically consist of three layers, tunica intima, tunica media, and tunica adventitia, from inside to outside. The middle layer is mostly comprised of smooth muscle cells. In Mjolnir, based on the cell phenotypes and the expression levels of the smooth muscle marker *MYH11*, we annotated 29 and 11 vessel internal regions on two post-LVAD heart tissues (Figures 4a and S19a-c). We extracted highly expressed genes in these vessels, finding 56 genes common to both tissues (Figures 4b and S19d). Excluding smooth muscle markers (such as *TAGLN*, *ACTA2*, *MYH11*, and *MYLK*), *PLA2G2A* stood out. *PLA2G2A* was reported to promote cell proliferation, angiogenesis, and tissue regeneration^48^ in several tumor types. In the cardiovascular field, another study showed that the PLA2G2A is specifically expressed in donor heart fibroblasts compared with the failing heart fibroblasts^49^. Our previous work highlighted fibroblasts’ role in revascularization^50, 51^, leading us to hypothesize that *PLA2G2A* expression is a signature of cardiovascular regeneration*.* To validate this hypothesis, we divided the vessel cells into *PLA2G2A*^*+*^ and *PLA2G2A*^*-*^ groups based on the distribution of *PLA2G2A* expression (Figure 4c). We found that upregulated genes in *PLA2G2A*^*+*^ cells were enriched in pathways including tube morphogenesis and blood vessel morphogenesis and development (Figure 4d). The expression levels of *PLA2G2A* in the vessels across two patients exhibited an apparent pattern: high *PLA2G2A* expression was linked to vessels surrounded by connective or adipose tissues while low expression was associated with vessels surrounded by myocardium (Figure S19e-f). Follow-up immunofluorescence staining of tissues from two post-LVAD patients further confirmed *PLA2G2A* presence in vessels at the protein level, supporting its role in heart recovery (Figure 4e). Altogether, Thor’s histology-transcriptome joint analysis revealed cell-resolution gene expression patterns and identified crucial molecules that may drive vascular regeneration in heart tissues.

### Thor enables multi-layered investigation of hallmarks in DCIS data

**Figure 5:**
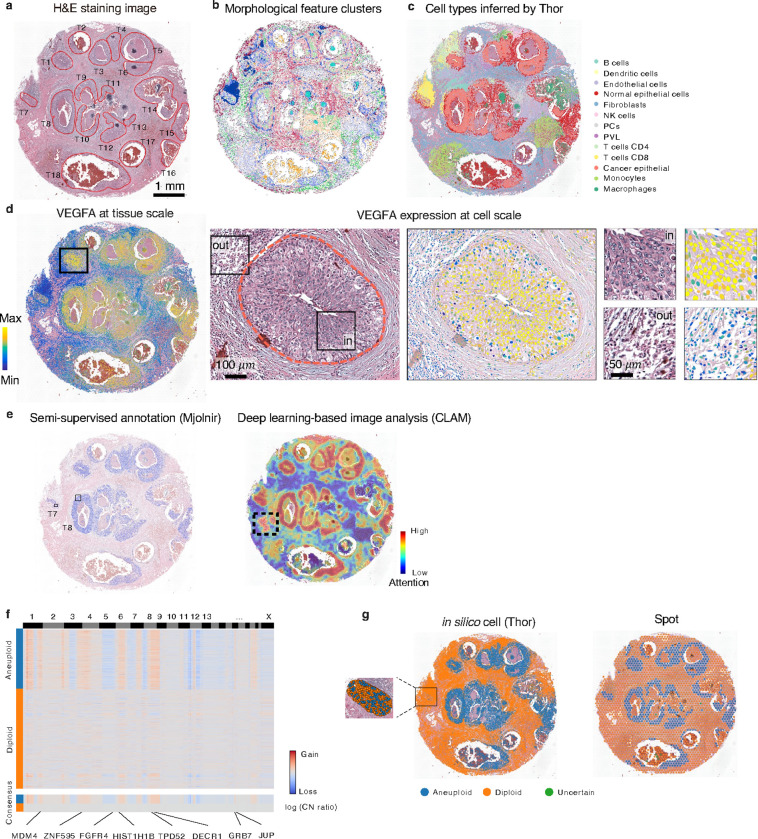
Thor provides unbiased screening of hallmarks in cancer. (a) H&E staining image of the DCIS tissue. The annotation of eighteen major tumor regions (T1-T18) in the DCIS tissue is adapted from the annotation by pathology experts (Agoko NV, Belgium). (b) Leiden clusters of the segmented cells using morphological features. The list of image features and details of Leiden clustering are provided in Supplementary Note 1. Colors represent cell clusters. (c) The spatial distribution of cell types. Cell types are obtained by Cell-ID using the Thor-inferred spatial transcriptome of the *in silico* cells and refined with cell type markers. (d) *VEGFA* gene expression pattern at tissue and cell scales in tumor region T1. (e) The tumor regions identified by high attention values in CLAM and semi-supervised annotation in Mjolnir. The black dotted square marks a high-attention region where adipocytes are predominantly located. (f) Heatmap of the copy number profiles inferred by CopyKAT based on the *in silico* cell-level transcriptome predicted by Thor. A selected list of breast cancer-related genes is provided. (g) Aneuploid (tumor) and diploid (non-tumor) regions inferred by CopyKAT show consistent results between *in silico* cell-level transcriptome and spot data.

Thor offers rich layers of information through streamlined multi-modal analyses within a unified platform. To showcase Thor’s strengths and functions, we analyzed a well-validated DCIS dataset that has been used as benchmarks widely^18, 52^. DCIS is a potential precursor to invasive ductal carcinoma, a condition that can progress into a form requiring surgical intervention and radiotherapy. Understanding the heterogeneity of various DCIS regions is crucial for elucidating the factors driving their diverse behavior. The DCIS dataset comprises 18 pathologist-annotated major tumor regions (T1-T18; Figure 5a). Histological features of segmented cells identified distinct clusters, underscoring their ability to distinguish between tissue regions (Figure 5b; Supplementary Note 1). Through integrated histological features and ST analyses, Thor enabled a multi-layered investigation of breast cancer hallmarks.

First, Thor facilitates cell type annotation at single-cell level. The spatial distribution of annotated cell types aligned with the results from state-of-the-art methods such as CytoSPACE and RCTD^18, 53^ (Figures 5c and S20). The signature genes of each cell type are provided in Supplementary Table S3 for reference. While these methods require scRNA-seq reference data, Thor overcomes the limitation by integrating the underused histological features with ST. Additionally, Thor’s advantage lies in providing gene expression for individual cells detected directly from the tissue image for additional analysis, maintaining cells’ spatial arrangement.

Second, Mjolnir enables interactive exploration of the spatial profiles of key molecules on the gigapixel histological images seamlessly at various zoom levels spanning from the whole tissue to the cellular scale. As an example, the visualization of *VEGFA*, a pivotal angiogenic factor influencing tumor growth and metastasis, highlighted distinct abundance levels within tumor subpopulations at the cellular resolution (Figure 5d). Additional gene expression profiles at both spot and *in silico* cell levels were provided in Figure S21. A closer examination of the tumor region T1 using Thor revealed the morphological features and the nuanced expression patterns of the cancer cells. *VEGFA* exhibited the highest expression at the center of the tumor region T1, gradually decreasing in abundance towards the boundary; and was minimally expressed in the myeloid cell population outside of T1.

Third, Thor enables efficient search of similar cells in the combinatory space of histological and transcriptomic features. We curated a small set of tumor cells in T8 based on cell morphology and the key gene expression profiles. Cells in most tumor regions were successfully identified (Figure 5e; accuracy: 0.83). Interestingly, hardly any tumor cells in T7 matched the curated set, likely due to its distinct immune microenvironment. Instead, tumor cells in T7 were effectively identified using a separate set of curated cells within T7 (Figure S22). This demonstrates Thor’s precision in identifying tumor cells through integrated analysis.

Using only the H&E image, the clustering-constrained-attention multiple-instance learning (CLAM) method^2^ identified high-attention regions (Figure 5e) that broadly overlapped with pathology-annotated tumor areas (Figure 5a). However, CLAM also identified adipose tissue as high-attention region, which was not directly relevant to cancer (black box in Figure 5e). These false positives happen for patterns which are not strongly represented in the negative samples^2^, and may require additional training of CLAM on curated datasets of labelled WSIs for more improved specificity. This demonstrated the value of tissue image analysis for tumor detection while highlighting the need for further multi-modal integration to reduce false positives.

Fourth, Thor’s cell-level molecular signature and pathway enrichment analysis provided deeper insights into the heterogeneity of tumor progression. By examining spatial patterns of oncogenes and tumor suppressors, we observed a marked contrast between *ERBB2* (*HER2*; an oncogene) and *ATM* (a tumor suppressor)^54^: *ERBB2* was highly expressed across all tumor regions, whereas *ATM* was upregulated exclusively in region T7 (Figure S23). An unbiased investigation of cancer hallmark pathways further highlighted their complexity across different tumor regions at the cell level, including DNA repair, a crucial process for maintaining DNA integrity and preventing mutations (Figure S24). Notably, despite the low expression of *ESR1* (Figure S21), the estrogen response pathway still showed significant enrichment in tumor regions (Figure S24), emphasizing the power of pathway-based analyses to refine breast cancer classification.

Lastly, genomic CNV inference from Thor’s cell-level transcriptome classified tumor and normal cells. Thor successfully uncovered genome-wide CNV profiles in DCIS (Figure 5f), achieving an F1 score of 0.78 and a Jaccard index of 0.64 (Figure 5g), which closely aligned with pathology-annotated tumor regions and surpassed spot-level CNV analyses (F1 score: 0.73; Jaccard index: 0.58; Figure 5g). Unlike spot-level CNV, which averages all cells in a spot, and can misrepresent regions containing both aneuploid and diploid cells, Thor’s single-cell approach accurately detected mixed populations, as exemplified by tumor region T7. Spot-level analysis labeled this entire region as aneuploid, whereas Thor-inferred and CytoSPACE-mapped single-cell data identified a mixture of aneuploid and diploid cells. Thor further revealed key copy number aberrations across all tumor cells, including gains in 1, 2q, 8q, 12p, and 18p and losses in 5, 8p, 11q, and 12q. These aberrations highlighted well-known breast cancer-associated genes, such as *MDM4*, *ZNF595*, *FGFR4*, *HIST1H1B*, *TPD52*, *DECR1*, *GRB7, and JUP*^55^. CNV analyses provide critical insights into the genomic alterations that underpin tumor heterogeneity and progression, offering potential biomarkers for prognosis and therapeutic targets. Altogether, Through Thor’s unified platform of integrated analyses of histology and transcriptomics data, Thor offers an unbiased, multi-layered view of breast cancer hallmarks.

### Thor reveals heterogeneity of immune response in tumor regions of DCIS

**Figure 6:**
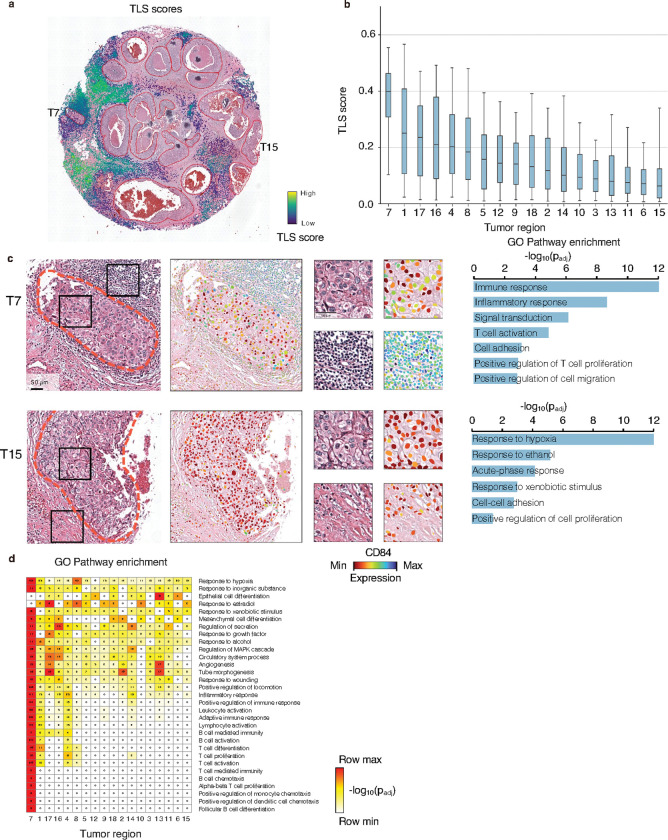
Thor reveals mechanistic insights into the immune response of DCIS. (a) Cell-level TLS scores. The TLS score is calculated based on 29 genes. (b) Boxplot of the TLS scores in the 18 tumor regions. The tumor regions are ranked according to the median TLS score. The middle line in the box plot, median; box boundary, interquartile range; whiskers, 5–95 percentile; minimum and maximum, not indicated in the boxplot. (c) Zoom-in view of the tumor regions with highest/lowest (T7/T15) TLS scores. The expression level of one DEG, *CD84*, is visualized in the inner and perimetral parts of the tumor regions. GO pathway enrichment is based on 300 up-regulated (fold change > 2, adjusted p_value < 0.01 using t-test) and 300 down-regulated (fold change < 0.5, adjusted p_value < 0.01 using t-test) DEGs between T7 and T15. (d) Heatmap of the GO pathway enrichment based on the up-regulated DEGs in each tumor region compared to the rest (fold change > 1.5, adjusted p_value < 0.05 using t-test).

We further investigated cell-level immune responses in DCIS by computing the well-established “TLS score” to quantitively capture local immune activity around the tumor regions^55, 56^. For each cell, the score was calculated by comparing the averaged RNA expression levels of 29 signature genes, including key markers of immune cells such as T cells, monocytes, macrophages, and fibroblasts^56^, to the average expression of randomized control genes (Figure 6a, see Methods). We then ranked tumor regions based on the median TLS scores of cells residing within each region, along with those in a narrow peritumoral layer (one spot-size outward from the tumor boundary). Regions T7, T1, and T17 exhibited the highest median TLS scores, indicative of robust immune activity (Figure 6b).

To gain deeper insight into the molecular distinctions of these high- and low-scoring regions, we performed differential gene expression analyses comparing tumor regions with the highest TLS scores (T7, T1, and T17) and those with the lowest (T11, T6, and T15). Several immune-related genes showed pronounced variation: for example, *CD84* and *SMAD3* were abundant in T7 but nearly undetectable in T15 (Figures 6c and S25), whereas *KANK1*, often relevant in cancer prognosis, was highly expressed in T6 and T15 but absent in T7. We further examined functional distinctions and interactions between tumor regions and their immediate peritumoral neighbors (Figure S25b). T7 was enriched for pathways linked to immune responses and T cell co-stimulation, whereas T15 was enriched for tumor-related pathways such as hypoxia response and cell adhesion.

Finally, we conducted unbiased region-specific pathway enrichment analysis based on upregulated genes in each tumor region (compared to the remaining tissue). As expected from the high TLS scores, T7-specific genes were linked to immune response, T cell activation, and inflammatory response pathways, while T15-specific genes were associated with hypoxia response and cell-cell adhesion (Figure 6c). A global heatmap (Figure 6d) illustrated that other tumor regions, such as T9, T14, and T13, also displayed strong enrichment for inflammatory and immune pathways. Notably, high-scoring regions like T7 and T1 showed enrichment of B cell activation pathway, suggesting more robust immune microenvironments that may be therapeutically relevant. By mapping these immune landscapes at single-cell resolution, Thor provided valuable insights into the functional heterogeneity among tumor regions, supporting a refined understanding of immune-tumor interactions in DCIS.

### Thor enhances gene expression imputation in high-resolution Visium HD data

Recent advances in spatial transcriptomics technologies are pushing toward cellular or even subcellular resolution, yet these high-resolution methods still face challenges such as substantial dropout and technical noise. To demonstrate Thor’s effectiveness under these conditions, we generated a high-resolution dataset from an in-house bladder cancer sample using Visium HD. In our experiment, despite the spatial resolution of up to 2 *μm* square bins (aggregated into 8 *μm* square bins for analyses as recommended by 10x Genomics), Visium HD data exhibited high technical noise. For example, *PTPRC* (a lymphoid marker) appeared sparsely distributed in immune-rich niches, while *SPINK1* (a urothelium-associated gene) was erroneously detected in non-tissue regions (Figure S26a). We applied Thor to integrate the 2 *μm* square bins with the histology image. Thor’s cell-level imputation yielded more coherent expression patterns than 8 *μm* square bins. the that correctly localized *PTPRC* to immune areas and *SPINK1* to the tumor boundary, aligning with pathology annotations.

Beyond single-gene assessments, Thor-imputed data captured distinct cell populations more accurately. For instance, cluster 7 in Thor’s results precisely matched the pathology-annotated immune cell regions (Figure S26b), whereas the raw bin-level data overestimated immune cell presence (Figure S26c).Similar overestimation of certain cell types was also reported recently in Visium HD data^57^. Taking together, these proof-of-concept analyses underscore Thor’s ability to refine gene expression signals and enhance biological interpretability in high-resolution ST datasets.

### Robustness of Thor to parameter settings

Thor is designed to be highly flexible, allowing customization of various parameters that control the preprocessing of image/transcriptome data, cell-cell graph construction, and the Markov diffusion process. To evaluate Thor’s robustness, we conducted a systematic sensitivity analysis of key parameters, including the diffusion step size t, the number of cell neighbors k, and the number of principal components nPC of the transcriptome data. Thor constructs a shared nearest neighbors (SNN) cell-cell graph based on the k-nearest neighbors in the combinatory space. First, we tested a range of k values on the MOB dataset while keeping other parameters fixed (t=40 and nPC=10). To reduce bias from highly expressed genes, we applied z-score normalization for each gene. We then calculated the Pearson correlation coefficients (r) across each pair of k settings. Thor demonstrated strong robustness for k values between 4 and 10, with a mean r=0.88 and standard deviation (std)=0.09. However, very small k values (<3) may produce disconnected cell graphs, whereas very large k values (40-100) may lead to over-smoothing and weaker correlations with the results of other k values (mean r=0.56, std = 0.27). Second, we evaluated the impact of varying nPC values while fixing k=5 and t=40. As shown in Figure S27a, Thor remains highly robust when nPC>=8 (mean r=0.94, std = 0.05). In contrast, nPC<4 fails to capture sufficient complexity in the data, leading to lower correlations with high nPC values. Third, we also evaluated a range of diffusion time t while keeping nPC=10 and k=5 fixed. Thor converged after approximately 10 diffusion steps, achieving a mean r=0.90 (std = 0.10) for t=10. However, large t values (e.g. t>50) may notably increase run time without significant performance gains (Figure S27b). Overall, our analyses show that Thor is robust to a broad range of ,k, and nPC values. These findings indicate that minor adjustments within reasonable parameter ranges have minimal effect on Thor’s results, which justifies that we kept a common set of parameters across all case studies.

Moreover, variational autoencoder (VAE) is widely used for RNA-seq data analysis^58–60^. Thor can utilize the latent representation in VAE for faster predictions. In the fast mode, the Markov diffusion is conducted on the VAE latent embeddings. The hyperparameter tuning, such as adjusting the input and latent dimensions of VAE can affect the results of Thor and contributes to generalizability. The input dimension should depend on the genes of interest, such as highly variable genes or spatially variable genes. Moreover, a proper latent dimension should sufficiently capture the biological complexity in the data. For instance, a latent dimension of 10 is set by default in scvi-tools^59^, with 20 or 30 being appropriate for more complex scRNA-seq datasets. We evaluated Thor’s performance on the MOB dataset by varying the latent dimensions in separate VAE models (8, 16, 20, 32, 64, and 128), while keeping other parameters fixed parameters fixed (nPC=10, k=5, and t=40). Thor-predicted gene expressions remained highly consistent with Pearson’s r>0.85 across all settings (Figure S27c). These results indicate that Thor is robust to a broad range of parameter settings.

## Discussion

Thor is an extensible and customizable platform detailed in the following aspects.

- The cell-level ST broadens the spectrum of downstream analyses to those originally designed for scRNA-seq data. Outputs from Thor are ready to be interfaced with a variety of existing libraries for analyses such as Squidpy^24^ and stLearn^25^ and can be easily adapted for scRNA-seq tools. Currently, Thor has included submodules such as cell-specific pathway enrichment^61^, inference of genomic CNV profiles^62^, and ligand-receptor analysis^63^.
- Thor supports customized cell features for building the cell-cell network. In this work, we highlight Thor’s performance using intensity-based morphological features such as color intensities of the staining image patches. The inclusion of more task-relevant features elevates the quality of the cell-cell network. For example, research has shown that spatial cellular graphs built from multiplexed immunofluorescence data enable the better modeling of disease-relevant microenvironments^64^. In addition, Thor supports direct input of a cell-cell network adjacency matrix.
- Beyond spatial transcriptomics, emerging omics technology such as spatial metabolomics and proteomics are increasingly adopted to capture local metabolic or protein-level processes that underlie key tissue functions and disease mechanisms. While our current work focuses on applying Thor to spatial transcriptomics, we envision that its underlying framework, which constructs a cell-cell graph from spot-level data, cell coordinates, and histological features, then refines those data through graph diffusion, could be adapted for spatial metabolomics or proteomics as well. By substituting transcriptomic values with metabolomic or proteomic intensities, Thor could enable a more comprehensive, multi-omic view of tissue biology at single-cell resolution. We anticipate that future developments will provide deeper insights into complex tissue characterizations by integrating these additional modalities.

Thor integrates histological features and transcriptomic features by inferring cell-level ST. Notably, Thor does not require any additional scRNA-seq data as a reference. This not only reduces the sequencing cost but also proves practically advantageous in FFPE tissues. FFPE tissues serve as the most abundant specimens for longitudinal studies with preserved tissue morphological details, yet RNA-seq profiling encounters hurdles due to RNA crosslinking, modifications, and degradation. The Visium platform offers a solution for profiling mRNA levels in both fresh-frozen and FFPE tissues, employing a de-crosslinking process^65^. Nevertheless, it falls short of providing cellular-level resolution. In contrast, commonly used methods like chromogenic immunohistochemistry (IHC) for assessing in situ biomarker expression in FFPE tissues are limited by the number of analytes, non-linear staining intensity, and the subjective nature of the quantitative analysis^66^. Thor strategically leverages the advantages of Visium and overcomes these challenges by delivering cell-level whole-transcriptome analysis, reducing the cost and workload.

Thor offers several advantages over existing frameworks for studying histological structures. PROST uses spatial relationships and transcriptomics data to identify spatially variable genes and to cluster spatial domains, but it does not enhance the resolution of the original ST data^21^. Thus, with Visium data, PROST operates at the Visium-spot level and does not utilize histology images. By contrast, Thor integrates cell-level features from histology images with spot-level transcriptomics, enabling inference of gene expression at the single-cell level and providing a more granular analysis of histological structures from Visium data. METI, meanwhile, is an end-to-end framework tailored to cancer ST data, mapping tumor cells and the surrounding microenvironment primarily in oncology-focused contexts^22^. Thor, on the other hand, was conceived as a more generalizable approach applicable across various tissues, disease states, and organisms. Moreover, neither PROST nor METI directly output single-cell gene expression. In contrast, Thor integrates histological and transcriptomic data in a task-agnostic manner to infer spatially resolved single-cell gene expression. This capability supports a broad range of downstream analyses and comes bundled with extensive analytical modules, including pathway enrichment, spatial gene module identification, differential gene expression, transcription factor activity estimation, and interactive whole-slide data visualization. Taken together, these features allow Thor to complement and extend the capabilities of frameworks by offering deeper spatial and molecular insights into tissue architecture.

Thor stands out as a comprehensive, user-friendly platform designed for multi-modal tissue analyses. As a model-based computational method, Thor operates efficiently on a laptop, presenting a practical advantage to existing deep learning-based approaches that demand abundant training data and intricate computational skills. The platform includes an interactive web-based tool Mjolnir, which enhances the analysis experience by allowing users to thoroughly investigate cell-level information through gigapixel histological images conveying various multi-modal attributes. Its intuitive interface enhances accessibility to a broad user base. Mjolnir incorporates a tile server algorithm that dynamically loads gigapixel images for smooth navigation. This not only resolves computational resource demands for visualization but also significantly improves the overall usability and responsiveness of the platform during analysis sessions, even on a laptop. Furthermore, Mjolnir functions as a standalone tool, offering users the flexibility to upload their images and cell-level attributes.

With rapid breakthroughs in deep-learning-based computer vision algorithms^7, 8, 67–69^, accurately detecting cell nuclei has become increasingly viable, transforming the challenge of cell detection in high-density regions^70, 71^. As an integrative spatial transcriptomics analysis platform, Thor incorporates multiple SOTA tools for cell segmentation and also supports manually/strategically added missing cells, enabling enhanced flexibility and adaptability for diverse workflows. Thor is designed to stay aligned with ongoing advancements in cell segmentation technologies, ensuring that its methods remain cutting-edge. Thor extracts tile-based image features from an image patch centered at the segmented cell nucleus centroid to capture the local environment surrounding the nucleus. These features are not limited to the nucleus itself but include the tissue context within the image tile, providing a comprehensive representation of the cell’s local environment from histology. Tile-based feature extraction is a practical strategy widely adopted in histology image analysis by deep-learning models and pathology foundation models. It facilitates a wide range of downstream tasks, including cell segmentation, cell type annotation, and tumor microenvironment profiling^15, 17, 72–75^. We recognize that certain cell types or microstructures may require more specialized descriptors. To address this, Thor provides an API (*thor.pp.image.WholeSlideImage.load_external_cell_features*) that supports morphological features generated by external tools (e.g., CellProfiler or CellViT). Researchers can extract customized metrics, such as cell shape, texture, or intensity profiles, then input these features into Thor, effectively augmenting or replacing the default cell detection and tile-based features. This flexible design allows Thor to accommodate a wide spectrum of histological analyses and cellular phenotyping tasks, ensuring that users can tailor the platform to their unique research objectives.

Recent advances in spatial transcriptomics technologies have pushed spatial resolution toward cellular or even subcellular level^27, 32, 76^, yet each technology still faces practical hurdles that Thor can help address. For instance, although Visium HD offers sub-cellular bin sizes, it can suffer from high dropout rates, low gene coverage^57^, and imperfect bin-to-cell alignment^77^ Meanwhile, Slide-seq may provide sparse transcript detection and limited capture size^78^, and image-based platforms such as Xenium and CosMX rely on predefined gene panels and may omit genes of interest. Our results show that by integrating Visium HD data with histological features, Thor reduces technical noise and reveals spatially coherent expression patterns that match pathology annotations. Beyond improving data quality, Thor functions as a comprehensive downstream analysis platform capable of handling the computational and visualization demands posed by large-scale ST datasets, where a single slide can contain millions of bins or hundreds of thousands of cells per slide. Thor’s interactive visualization tool, Mjolnir, renders gigapixel images and facilitates responsive exploration on standard computing hardware. Moreover, Thor remains a cost-effective option for achieving single-cell level analyses with standard Visium data (approximately 70% less expensive than Visium HD), benefiting labs with resource constraints or those seeking to reanalyze legacy ST datasets.

Thor has several limitations. First, it relies on high-resolution histology images (typically 0.25 to 0.5 *μm* per pixel) for cell detection and histological feature extraction. Real-world scenarios may introduce complexities, such as loss of focus in imaging or improper staining across large tissue regions. Such conditions may lead to missed cell detection or unrepresentative image features and Thor’s performance may understandably suffer. Additionally, Thor’s performance may be affected in regions where nuclei are difficult to identify, such as cells in peripheral areas with flat nuclei. Incorporating higher precision imaging techniques, such as the DAPI imaging used in the Xenium data cell detection, could help address this issue. Second, Thor does not currently support multi-sample integration, as batch effects in transcriptomics data and histological variability between tissue sections introduce biases that complicate direct comparisons and spatial alignment. These challenges also limit the applicability of semi-supervised annotation across multiple samples. Third, Thor does not operate at subcellular resolution to provide further finer level analysis. Due to the light diffraction limits in standard histology imaging and complex morphological variability of subcellular structures, robust and accurate segmentation of individual organelles or subcellular structures are highly challenging^79^ and restricted for certain organelles in restricted platforms^69, 80, 81^. Thor’s cell-level integrated analyses of transcriptomics and histology may complement with nanoscale spatial omics technologies. Its modular architecture could, in future work, integrate with subcellular methods like Stereo-seq to bridge tissue, cellular, and subcellular-level insights.

In conclusion, Thor effectively leverages ST analysis by integrating histology and transcriptomics, refining gene expression to the single-cell level and enabling more precise characterization of tissue architecture. This approach provides a valuable foundation for future cross-modal integration, including highly multiplexed imaging techniques (e.g., CODEX or MIBI) to achieve a more comprehensive, multi-modal understanding of spatial -omics data. By enabling the exploration of cellular interactions across spatial landscapes, Thor not only facilitates discovery of biological insights but also lays the foundation for the development of novel therapeutic modalities, thereby advancing the field of precision medicine for more effective and personalized patient care.

## Methods

### Overview of Thor

Thor integrates transcriptomics and histological information by faithfully inferring the whole transcriptome of *in silico* cells. Thor does not require training for the inference of cell-level gene expression. Instead, it operates per slide through a four-step modularized workflow (see Supplementary Note 1).

- Identify cells and extract locations and morphological features of each cell in their spatial neighborhood from the histological image. Meanwhile, the ST data is preprocessed, and the gene expression of the cells is initialized to their nearest spots.
- Compute multi-modal distances between cells and construct the cell-cell network based on their morphological features, geometrical locations, and the transcriptome collectively.
- Convert the distances to affinities using an exponential kernel, so that the similarity between two cells decreases exponentially with the multi-modal distance.
- Infer gene expression of the cells by transitioning information flow between similar cells and prohibiting that from cells covered by a heterogeneous spot.

Then the predicted cell-level gene expression can be applied to perform downstream analyses including interactive analysis in ROIs. The modules are described in detail as follows.

### Nucleus segmentation and feature extraction from histological images (reordered for its importance)

Nucleus segmentation is critical in the analysis of histological images, enabling quantitative assessment of the number of nuclei, density, and morphological characteristics. Thor integrates several state-of-the-art tools^6, 7, 67^ for nucleus segmentation and supports user-supplied segmentation results to ensure adaptability across diverse platforms.

For jointly analyzing the histological image and the transcriptomics, Thor employes two filtering processes: Thor eliminates out-of-context nuclei by superimposing segmented nuclei on the aligned spatial spots and removing nuclei whose centers are beyond a cutoff distance from the nearest spots. The default cutoff distance is the diameter of the spots. Furthermore, Thor detects and removes isolated cells or artifacts located away from the tissue boundaries.

Tile-level histological features are extracted to represent the local environment surrounding each cell. This local environment is defined by extending from the nucleus centroid to a given distance, typically twice the mean distance between the nearest nuclei centroids. In this study, we included image features such as the mean and standard deviation of color intensities, as well as image entropy, within a defined radius around each nucleus on the tissue. These features have proven to be effective in constructing cell-cell networks for Thor inference across all tested datasets. Additionally, Thor supports custom functions for feature extraction and allows the integration of user-supplied nucleus or cell-specific features, as well as deep-learning-derived features, offering flexibility and extensibility in the analysis process. Thor is designed to incorporate advancements in segmentation toolkits to stay on the front of cell segmentation field.

### Constructing the cell-cell network

Thor infers cell-level gene expression based on the cell-cell network. Connectivity between cells is determined by their distances in the combinatory feature space, formed by morphological features, geometrical locations, and the low-dimensional representation of the transcriptomic data. The features are standardized to normal distribution N(0,1) across all the cells. Nearest neighbors are included to construct the KNN cell-cell network based on the distance metric $d_{ij}$ in the feature space, i.e.

(1) $$d_{ij}=\sqrt{{\left(w^{gen\_m}d_{ij}^{gen}\right)}^{2}+{\left(w^{geo\_m}d_{ij}^{geo}\right)}^{2}+{\left(d_{ij}^{mor}\right)}^{2}}$$

where $d_{ij}^{gen}$, $d_{ij}^{geo}$, $d_{ij}^{mor}$ are the dimension-normalized Euclidean distances in the transcriptomic (reduced dimension), geometrical, and morphological feature space, respectively; $w^{\mathrm{gen\_m}}$ and $w^{geo\_m}$ are the respective weights in relative to the morphological feature distance. Increasing the $w^{\mathrm{geo\_m}}$ value leads to a more localized network and increasing the $w^{\mathrm{gen\_m}}$ value favors the distance in the transcriptomic space.

Next, to preserve local structure and account for the non-uniform density of the cells, the KNN cell graph is converted to a shared nearest neighbors (SNN) graph. SNN prioritizes connections among cells that have multiple neighbors in common. This emphasis can unveil intricate data patterns and has demonstrated a reduced susceptibility to isolated noisy data points. Cells i and j are connected if the proportion of their shared neighbors $w_{ij}$ is beyond a given threshold.

(2) $$w_{ij}=card(NN(i)\cap NN(j))/k$$

In Eqn. (2), NN(i) and NN(j) refer to the sets of nearest neighbors of cell i and cell j, respectively. card refers to the cardinality of the overlap set. k is the number of nearest neighbors considered.

### Feature-preserving Markov diffusion model

As the ST spot data represents the aggregate expression across enclosed cells, we hypothesize that gene expression in a homogeneous spot is more accurate compared to a heterogeneous spot. The heterogeneity of a spot is quantified by the coefficient of variation in cellular features of all cells mapped to the spot or by the Shannon entropy when cell type labels are available (e.g., from spot deconvolution methods). On the SNN cell graph, Thor ensures that more accurate gene expression data corrects the less accurate ones while inhibiting the propagation of the less accurate information through modulation of node weights and edge weights.

Cells mapped to a more homogenous spot carry a larger node weight $G_{i}$, thus more robust to variations. $G_{i}$ is calculated as an exponential kernel on the heterogeneity of the corresponding spot.

(3) $$G_{i}=e^{-kS_{i}}$$

where k is the inverse kernel width that controls the shape and $S_{i}$ is the heterogeneity of the spot enclosing cell i. The edge weight $\epsilon_{ij}$ between two cells i and j is computed as the product of the “bandwidth” $w_{ij}$ as shown in Eqn. (5), proportion of their shared neighbors defined in Eqn. (2), and the “latency” $L_{ij}$ defined in Eqn. (4).

(4) $$L_{ij}=\frac{1}{1+e^{-\alpha (G_{i}-G_{j})}}$$

(5) $$\epsilon_{ij}=\left(1-\delta_{ij}\right)L_{ij}{\;W}_{ij}+\delta_{ij}G_{i}$$

where α controls the steepness of the scaled sigmoid function.

The transition matrix $F_{ij}$ is then computed as,

(6) $$F_{ij}=(1-\lambda )\delta_{ij}+\lambda \epsilon_{ij}=\delta_{ij}-\lambda \left(\delta_{ij}-\epsilon_{ij}\right)$$

where the constant 1-λ∈(0,1) is the probability of keeping the original (self) gene expression. As shown in Eqns. (4–6), the “latency” is a key parameter that turns the symmetric SNN into an asymmetric network in favor of incoming information flow in a connection from the cell with a lower heterogeneity score, or a larger node weight, to the cell with a higher heterogeneity score.

The transition matrix takes the same form as in a Laplacian smoothing method, likewise, the diffusion causes shrinking in the transcriptome space. Therefore, we employ a well-known feature-preserving technique in the field of surface smoothing^82^ and introduce a reversed diffusion transition matrix $R_{ij}$ after the forward diffusion to inflate the transcriptome space.

(7) $$R_{ij}=(1-\mu )\delta_{ij}+\mu \epsilon_{ij}=\delta_{ij}-\mu \left(\delta_{ij}-\epsilon_{ij}\right)$$

where μ∈(-1,0), $\delta_{ij}$ is the Kronecker delta. In practice, the absolute value of μ is set marginally larger than λ for sufficient inflation.

The feature-preserving diffusion is composed of a forward diffusion step followed by a reversed diffusion step. Therefore, the effective transition matrix is computed as the matrix multiplication of the reversed diffusion transition matrix $R_{ik}$ and the forward diffusion transition matrix $F_{kj}$,

(8) $$T_{ij}=\sum_{k}R_{ik}F_{kj}$$

The resulting Markov transition matrix $F_{ij}$ represents the probability distribution of transitioning from each cell to every other cell in a single step. The transition matrix $T_{ij}$ is normalized by rows to ensure that the probabilities of incoming signals sum up to 1.

Lastly, after obtaining the Markov transition matrix, Thor performs graph diffusion to infer the gene expression at the cellular level.

(9) $$x^{n}=(T{)}^{n}x^{0}$$

where $x^{0}$ is the input gene expression initialized by the nearest spot-level values, $x^{n}$ is the final inferred gene expression, F is the feature-preserving Markov transition matrix, and n is the number of diffusion steps. The Markov diffusion converges rapidly, typically within 10 steps^83^.

Due to the substantial number of *in silico* cells, the diffusion can take hours. To speed up Thor, the Markov graph diffusion may be performed on the reduced-dimensional embedding, such as the latent variables of a variational autoencoder (VAE), and the transcriptome can be reconstructed from the latent variables. Finally, Thor rescales the gene expression to the same range as the input spot-level gene expression, and optionally samples cell-level gene expression considering stochasticity in scRNA-seq reads.

### Advanced analyses and dynamic visualization

Technical challenges arise when analyzing and visualizing systems containing a vast number of cells. The WSIs are gigapixel-scale and typically encompass from 10,000 to 100,000 in silico cells within a 6.5 mm × 6.5 mm tissue sample. These large-scale datasets present significant difficulties in terms of computational resources and effective data visualization. To address these challenges, we adapted existing pipelines for analysis of cell-level multi-omics and imaging data, as well as developed a dedicated tool Mjolnir for interactive visualization of large biomedical images. Details for dynamic visualization and advanced analyses are as follows,

#### • Interactive visualization of histology and genomics.

Mjolnir leverages image-tiling technologies used by Google Maps, enabling seamless navigation through gigapixel images at a range of zoom levels. Mjolnir empowers users to visualize segmented components, including spots and cells/nuclei color-coded by gene expression or additional attributes, such as copy number profiles.

#### • ROI selection.

Mjolnir supports drawing and editing regions of any shape on the staining image. A user can export the selected ROIs in common data formats such as annData for gene expression, TIFF for image patches, and JSON for polygon coordinates, facilitating further analyses.

#### • DEG analysis.

Differentially expressed genes (DEGs) are extracted between two specified groups of cells. Thor treats individual cells in a group as replicates and assesses the significance of changes in gene expression using statistical models.

#### • Pathway enrichment analysis.

A pathway is represented by a group of specific molecules that collectively carry out vital functions within cells and organisms. Thor adapts the Python package *decoupler*^61^ to compute the cellular enrichment of pathways.

#### • TF activity analysis.

The activity of a TF is inferred by the expression levels of its regulated genes. Thor adapts *decoupler*^61^ to compute cellular TF activity.

#### • CNV analysis.

Thor integrates the R package CopyKAT^62^ for CNV analysis with a wrapper function. Thor expedites the calculation of CNV by parallel computing.

#### • TLS score.

The TLS score is calculated based on 29 signature genes, including markers of immune cells such as T cells, monocytes, macrophages, and fibroblasts^56^. The TLS score in the DCIS dataset was calculated with the scanpy.*tl.score_genes* function in SCANPY^84^, as the averaged expression of a set of genes subtracted by the averaged expression of a set of randomly sampled genes.

#### • Cell-cell communication.

Thor integrates the python package COMMOT^63^ to analyze cell-cell communication, which accounts for competition among different ligand and receptor species as well as spatial distances between cells. Thor boosts the calculation by implementing a more efficient function to compute the cell-cell spatial distance matrix within the interaction cutoff distance in place of the original implementation.

### Post-LVAD heart failure ST data collection

#### Sample collection and preparation

Tissues were collected from patients wearing LVAD before a heart transplant. All samples were obtained under an approved IRB protocol (Pro00006097:1 Congestive Heart Failure) at Houston Methodist Hospital. FFPE heart failure tissue samples were collected using standard-of-care procedures. Tissue sections (10 *μm*) obtained from the FFPE tissues were mounted on Visium spatial gene expression slides (10x Genomics, 1000520). The samples were processed as described in the manufacturer’s protocols.

#### ST by 10x Genomics Visium

The tissue slides were permeabilized at 37 °C for 6 min, and polyadenylated mRNA was captured by oligonucleotides bound to the slides. Reverse transcription, second-strand synthesis, complementary DNA (cDNA) amplification and library preparation proceeded using the Visium Spatial Gene Expression Slide & Reagent Kit (10x Genomics, 1000520) according to the manufacturer’s protocol. After evaluation by real-time PCR, cDNA amplification included 13–14 cycles. Indexed libraries were pooled equimolarly and sequenced on a NovaSeq X Plus instrument in a PE28/150 run (Illumina). An average of 26, 011 paired reads were generated per spot and the median genes per spot were 2,277. Tissues were stained with H&E, and slides were scanned on a Pannoramic MIDI scanner (3DHISTECH) using a ×20, 0.8-NA objective.

#### Spatial profiling of vascular protein

To capture the spatial expression of the candidate protein, we adapted an established protocol for spatial mapping using immunofluorescence staining. This technique provides detailed visualization of gene expression within tissue contexts, allowing for precise localization and analysis of the candidate gene’s expression patterns across different tissue regions. First, paraffin-embedded sections were deparaffinized with xylene thrice for 5 minutes each. The sections were then rehydrated through a series of ethanol washes: twice in 100% ethanol for 2 minutes each, twice in 95% ethanol for 2 minutes each, and once in 75% ethanol for 2 minutes. The slides were then rinsed in ultra-pure water for 5 minutes, followed by Tris-buffered saline (TBS) containing 0.0025% TritonX-100 for 5 minutes. For antibodies recognizing surface proteins, a rinse with 1xTBS alone was used. Subsequently, the slides were subjected to antigen retrieval by placing them in a sodium citrate solution heated to 85°C on a hot plate for 10 minutes. The sections were then encircled with a pap pen, and the primary antibody was applied overnight in a dark, humidified chamber at 4°C. The following day, the slides were washed twice with either 1xTBS containing TritonX-100 or 1xTBS alone, depending on the nature of the protein of interest. Next, the slides were incubated with the secondary antibody in a dark chamber for 30 minutes. After incubation, the slides were washed twice and mounted using a DAPI-containing mounting medium. Microscopy images were obtained using an Olympus FV3000 Confocal microscope. A negative control slide was used to establish the threshold settings, which were consistently applied to all slides for image acquisition.

### Preprocessing the histology images

In order to accurately detect cells/nuclei from histology images, preprocessing steps including image normalization and augmentation of the histology images in this study adhered to recommending settings the cell segmentation tools. For StarDist, pixel values were clipped at 1% and 99.8% for all the (red, green, blue) color channels, and the trained model ‘2D_versatile_he’ was used. Cellpose internally included data normalization in the neural network. Following the recommendations in Squidpy, we inverted the color values of the H&E images and used the blue channel for nuclei segmentation^24^.

For the MOB dataset, nuclei were segmented from the H&E staining images with Cellpose^7^ using the parameters (*min_size = 10, flow_threshold = 0.4, channel_cellpose = 0*). For the human MI datasets, the 10x Genomics human breast cancer Xenium & Visium datasets, the 10x Genomics human DCIS dataset, and the post-LVAD human heart tissues, StarDist^67^ was used with the default parameters (*prob_thresh* = *0.05, nms_thresh* = *0.2*). For the mouse brain MERFISH dataset, the cell segmentation downloaded along with the data was used.

### Preprocessing the spatial transcriptomic data

The initial preprocessing steps involved quality control and library size normalization, adhering to the SCANPY standard protocols^84^. Highly/spatially variable genes were identified by established protocols^84–86^ for inference and following downstream analyses. A low-dimensional representation was obtained through dimension reduction methods, including PCA, UMAP, or by utilizing the latent space of a VAE.

### Parameter settings in Thor inference

Thor inference demonstrated robust performance to the variations in parameter settings. Therefore, in all analyses of this study, default parameters in Thor were employed, with specific configurations as outlined below. The construction of the cell neighborhood graph utilized an initial k-nearest neighbor approach, setting the number of neighbors to 5 (*n_neighbors = 5*). Additionally, the probability of retaining the original (self) gene expression, denoted as 1 − *λ* in Eqn. (6), was set to 0.2 (equivalently in Thor, *smoothing_scale = 0.8*), and the total number of diffusion steps was specified as 20 (*n_iters = 20*).

### CytoSPACE

CytoSPACE was performed on the Human ductal carcinoma in situ by Visium dataset to map single cells from a reference scRNA-seq data. A breast cancer scRNA-seq atlas by Wu et al. was used as the reference^87^. Default parameters were used. The default cell type information from the original study^87^ was projected.

### RCTD

RCTD was ran on the Human ductal carcinoma in situ by Visium dataset to deconvolute the cell type proportions in the spots. An annotated breast cancer scRNA-seq atlas by Wu et al. was used as the reference data^87^. Default parameters were used.

### iStar

For iStar prediction of superpixel level gene expression on the Human breast cancer by Visium data, default settings recommended in the documentation in the GitHub repository (https://github.com/daviddaiweizhang/istar) were used. We applied iStar to the post-Xenium H&E image and the paired Visium dataset. We set the desired pixel size to 0.25 *μm* for high-resolution inference of the spatial gene expression.

### BayesSpace

For enhancing spatial features on the simulation data, we set the number of clusters to the ground truth number of clusters, number of PCA components to 10, and spatial-enhancing Markov chain Monte Carlo (MCMC) rounds to 50,000. For enhancing spatial features on the mouse MERFISH-generated spot data, we set the number of clusters to 8, number of PCA components to 10, and spatial-enhancing MCMC rounds to 50,000. For enhancing spatial features on the 10x human breast cancer by Visium dataset, we set the number of clusters to 6 (the number of major clusters identified in the reference Xenium dataset), number of PCA components to 10, and spatial-enhancing MCMC rounds to 50,000. Other parameters were set to their default values.

### Cell type annotation by Cell-ID

Cell-ID was used to transfer the cell type information from an annotated reference scRNA-seq data to annotate single-cell data inferred by Thor^35^. Cell-ID was performed by a per-cell assessment in the query dataset evaluating the replication of gene signatures extracted from the reference dataset.

We followed the Cell-ID vignette and used the default parameters. For the MOB dataset from 10x Genomics, we used the cell type signatures from the scRNA-seq data^88^. For the human DCIS dataset from 10x Genomics, we used the cell type signatures from the scRNA-seq data (https://drive.google.com/file/d/1G8gK4MxCmRG4JZi588wloMsP8iZlQf_z/view?usp=share_link) for Cell-ID annotation^18^. We further refined the annotations by using expression levels of key gene signatures including *EPCAM* and *CDH1*, to distinguish between normal and tumor epithelial cells. Similarly, monocytes and macrophages were separated by using marker genes *VCAN* (versican) and *CD14*, which were upregulated in circulating monocytes and reduced upon differentiation to macrophages (Figure S20a).

### Pathway enrichment analysis

Functional enrichment analysis was performed using the over-representation analysis (ORA) method implemented in the Python package *decoupler*^61, 89^. For each cell, the top expressed genes were treated as the set of interest. For a given gene set (e.g. a GO term), a one-sided Fisher exact test was applied to test the significance of overlap between the gene sets. The resulting p values were log-transformed to yield enrichment scores, where higher scores indicate greater significance. For example, T cell proliferation score was calculated by overlapping the top expressed gene lists in each cell with the GO term positive regulation of T cell proliferation (GO:0042102).

### Transcription factor activity inference

The database CollecTRI and Python package *decoupler* were used for the TF activity inference. CollecTRI is a comprehensive resource comprising weighted transcriptional regulatory networks of TF-target gene interactions^90^. TF activities were estimated using the univariate linear model method implemented in *decoupler*^61^, by predicting gene expression levels based on the TF-Gene interaction weights from CollecTRI. The resulting TF activity scores provide directional insights: positive scores indicate active TFs driving gene expression, whereas negative scores suggest inactivity or repression.

### Gene module identification

Hotspot^36^ was used for identification of informative genes in the single-cell level spatial transcriptome dataset. For the module assignment by Hotspot in the MOB dataset, the number of nearest neighbors was set to 30 for creating the KNN graph. A false discover rate (FDR) cutoff of 0.05 was applied, thereby grouping 1,688 out of all the 2,781 highly variable genes into 8 modules.

### Datasets and preprocessing

#### Human breast cancer by Xenium & Visium:

The Xenium gene expression matrix and the Visium raw reads were downloaded from the 10x website (https://www.10xgenomics.com/products/xenium-in-situ/preview-dataset-human-breast). We mapped the Visium reads to the post-Xenium H&E staining image (In Situ Sample 1, Replicate 1) using 10x Space Ranger software (v2.1.0) for direct comparison between Thor-inferred result and Xenium data. The processed Visium gene expression matrix of the 306 genes, found commonly in the Xenium and the Visium datasets, and the post-Xenium H&E image were utilized as input for Thor/iStar/TESLA/BayesSpace. The Xenium data was employed as a reference for assessing the performance of Thor or iStar and was excluded during prediction.

#### Human ductal carcinoma in situ by Visium:

The gene expression matrix and the paired full-resolution H&E staining image were downloaded from the 10x website (https://www.10xgenomics.com/resources/datasets/human-breast-cancer-ductal-carcinoma-in-situ-invasive-carcinoma-ffpe-1-standard-1-3-0). The gene expression matrix was preprocessed and log-normalized expression of 2,748 highly variable genes was used to train a VAE network for accelerating Thor inference. The dimension of the latent space of VAE was set to 20.

#### Human healthy heart sample by Visium:

The full resolution H&E staining images of 12 samples were downloaded from links (https://www.heartcellatlas.org/) in the original publication^37^. The gene expression matrices and spot level expert annotations were provided in the annData files. The sample IDs include “HCAHeartST11702008” (vessel: S1), “HCAHeartST12992072” (vessel: S2), “HCAHeartST9383353” (vessel: S3), “HCAHeartST11290662” (node: S4), “HCAHeartST11702008” (node: S5), “HCAHeartST11702009” (node: S6), “HCAHeartST13228106” (adipose: S7), “HCAHeartST9383354” (adipose: S8), “HCAHeartST13228103” (adipose: S9), “HCAHeartST13228106” (fibrosis: S10), “HCAHeartST11350377” (fibrosis: S11), and “HCAHeartST8795936” (fibrosis: S12).

#### Human myocardial infarction by Visium:

The gene expression matrices and paired full-resolution H&E staining images of six samples were downloaded from links provided in the original publication^40^ (https://zenodo.org/records/6580069#.ZHYP9OzMK3I). Samples “10X0025” (RZ1), “ACH0019” (RZ2), “ACH0012” (IZ1), “ACH0014” (IZ2), “ACH008” (FZ1), “ACH006” (FZ2) were downloaded for analysis. To facilitate the comparison of tissues from ischaemic, fibrotic, and remote zones, where the expressed genes exhibited substantial variations, we aimed to maximize the overlap of genes among the six samples. After filtering out genes not expressed in any spot, we inferred the expression of all the remaining genes.

#### Human post-LVAD heart failure by Visium:

The gene expression matrices were obtained by using Space Ranger (v2.1.0), referencing the GRCh38 human Genome. The gene expression was preprocessed following SCANPY standard protocols. Log-normalized expression of all expressed genes was used as input.

#### Human bladder cancer by Visium HD:

The gene expression matrices were obtained by using Space Ranger (v2.1.0), referencing the GRCh38 human Genome. Gene expression matrices of the 2 *μm* square bins and 8 *μm* square bins were preprocessed using SCANPY. Log-normalized expression of highly variable genes of 2 *μm* square bins was used as input. Log-normalized expression of 8 *μm* square bins were used for comparison.

#### Mouse olfactory bulb by Visium:

The gene expression matrix and paired full-resolution H&E staining image were downloaded from the 10x website (https://www.10xgenomics.com/resources/datasets/adult-mouse-olfactory-bulb-1-standard-1). The gene expression was preprocessed following SCANPY standard protocols. Log-normalized expression of highly variable genes was used as input. A VAE network was trained to allow inference in the latent space. For evaluation, we downloaded the ISH images of selected genes in MOB from Allen brain atlas^33^ and the gene expression data from the Stereo-seq study^27^.

#### Mouse brain by MERFISH:

We used the Vizgen MERFISH mouse brain receptor map dataset that contains a MERFISH measurement of a 483 gene panel. Sample Slice 2 Replicate 1 was used and downloaded from https://info.vizgen.com/mouse-brain-map?submissionGuid=5606514b-5a81-4405-999e-327f908281cc. The DAPI image “mosaic_DAPI_z2.tif” was used for extracting image features of single cells. 8,597 cells in the hippocampus region were extracted (Figure S4a). After preprocessing, the log-normalized expression in 535 synthetic spots and the DAPI image features were used as input for Thor.

### Simulation details

Thor’s accuracy, sensitivity, and limitations of Thor were evaluated on simulated ST data under conditions, including diverse sources of ground truth data, variation in spot sizes, missouts in cell identification, false connections in a cell-cell network, and technical dropouts in sequencing. We extracted the positions of 6,579 cells in a mouse cerebellum Slide-seq data^32^, including the Granular (Cluster 1), Oligodendrocyte (Cluster 2), and Purkinje (Cluster 3) cells. Those cell locations reliably reflect the spatial distribution of cells in the real tissue and the gene counts in the single cells were simulated using Poisson distributions. We simulated a single-cell ST dataset by generating 1,000 genes of distinct spatial expression patterns, acting as markers for the three cell types. This included 350 genes for each of the first two cell types and 300 genes for the third cell type. Specifically, for the marker genes, the mean values of the Poisson distributions (λ) were randomly sampled in the range of (100, 200); and for the non-marker genes, λ values were randomly sampled in the range of (10, 20). Spots were then created on a grid and the spot-resolution gene expression levels are aggregated values of the enclosed cells.

- To assess the effect of different spot sizes, we simulated a series of spot diameters ranging from 25 to 150 *μm*, with nearby spots separated by 100 *μm*.
- To assess the effect of cell missouts, we randomly dropped 10%, 20%, 30%, and 40% of the cells.
- To assess the effect of the false connections in the cell-cell network, we added randomized connections in the cell-cell network, until 10%, 20%, 30%, and 40% of the cells contained randomized connections. In this evaluation, we did not directly use an image, instead, the cell positions were predefined, and the cell types were converted to one-hot vectors as image features. These features, combined with the generated spot-level gene expression, constituted the input for Thor, using default parameters to infer the cell-level ST data.

Additionally, to systematically assess the effect of technical dropouts, we simulated single-cell gene expression using the R package Splatter^91^ with no dropouts and with variable levels of dropouts. Splatter models the probability of transcript dropouts using a logistic function based on the mean expression levels $P_{\text{dropout}}(x)=1/\left(1+e^{-k*\left(x-x_{0}\right)}\right)$, where x is the mean expression level. The probability of transcript dropouts are controlled by two parameters, the midpoint parameter ($x_{0}$ or dropout.mid) and the shape parameter (k or dropout.shape). The former is the expression level at which 50% cells are zero, and the latter controls how quickly the probabilities change from the midpoint. To simulate a wide range of dropout conditions, we used combinations of dropout.mid values [1, 2, 3, 4, 5] and dropout.shape values [−1, −2, −5], with percentages of zero reads up to 63%. This allowed comprehensive assessment of the impact of varying dropout levels on Thor’s performance. Spot-level gene expressions generated from these single-cell simulations with dropout were then used as inputs for Thor.

We employed the Silhouette coefficient and Calinski-Harabasz index to measure the separation of clusters. The scores were calculated on the PCA embeddings of the corresponding gene expression arrays using the functions from the library *scikit-learn*. We randomly sampled 3,000 cells (without replacement out of all the 6,579 cells) 10 times in the calculation of the mean Silhouette coefficients for statistical significance.

The MERFISH data of the mouse brain receptor map consists of 83,538 cells and 483 genes. We simulated Visium-like spot-level data by creating a grid of evenly spaced “spots”. The molecule counts in a synthetic spot were aggregated over all the cells covered by the “spot”. The spot size was set to 100 *μm* and a total of 4,870 spots were simulated. We focused on the hippocampus region (Figure S4a), which consists of 535 spots covering 8,597 cells. For visualization purposes, major Leiden cell clusters in the original data were annotated according to the cellular locations in the hippocampus components and a previous study^19^. Minor cell clusters were merged and labeled as “Others”. A DAPI image in the dataset and the generated spot-level gene expression were jointly analyzed by Thor. For comparison, the positions of cells segmented from the source were used as our cell positions. Image features including the mean and standard deviation of the grayness and the entropy of image patches surrounding the cells were calculated. The predicted transcriptome and the ground truth transcriptome were integrated by harmony^92^.

### Evaluating cell-level spatial gene expression prediction accuracy

We used the root mean square error and structural similarity index to quantify the prediction accuracy for each gene. In the simulation datasets, NRMSE was employed to calculate the mean deviation of the predicted gene expression from the ground truth data in all cells, as defined in Eqn. 10.

(10) $$\text{NRMSE}\overset{def}{=}\frac{\sqrt{\frac{\sum_{i=1}^{n}{\left(x_{i}^{\text{pred}}-x_{i}^{\text{truth}}\right)}^{2}}{n}}}{\frac{\sum_{i=1}^{n}x_{i}^{\text{truth}}}{n}}$$

where $x_{i}^{\text{pred}}\left(x_{i}^{\text{truth}}\right)$ is the predicted (ground truth) gene expression in cell i; and n is the number of cells.

To assess the performance of predicted gene expressions by Thor and other methods, we calculated two pixel-centric metrics including SSIM, RMSE (pixels) and a cell-centric metric RMSE (cells). For pixel-centric metrics similar to the methodology from the iStar study^17^, both the ground truth and predicted gene expression were treated as grayscale images. Considering spatial contexts within the images, we calculated SSIM between the spatial structures of the ground truth and predicted gene expression images. Practically, we observed slight local distortions and shifts existed between the Visium and Xenium slides. For a more reliable measure of the prediction quality prediction, we therefore calculated the Complex Wave SSIM, which is insensitive to consistent spatial translation^93^. SSIM values range from 0 to 1, with 1 indicating identical images and 0 indicating no similarity. For cell-centric metrics, we aggregated the nearby gene expression of super-pixels to the ground truth cells.

### Comparison between Thor results and pathology annotation

To quantitatively assess Thor’s semi-supervised annotation function, we compared Thor against spot-level expert annotations. Because Thor assigns labels at the single-cell level, we employed majority voting to map these cell-level annotations to each spot. This allowed a direct comparison with expert-labeled spots. We report two commonly used classification metrics, accuracy, and area under the curve (AUC) as defined below. Accuracy is defined as the ratio of correct predictions to the total number of predictions. The area under the receiver operating characteristic (ROC) curve, which plots the true positive rate (sensitivity) against the false positive rate (1 – specificity) at various threshold settings. A higher AUC suggests better overall classification performance.

To quantitively assess Thor’s prediction of aneuploid cells through CNV analysis, we compared against pathology-annotated tumor regions. We used two metrics F1 score and the Jaccard index. F1 score is calculated as the harmonic mean of precision and recall, and a higher F1 score indicates a better balance between precision (the proportion of predicted positives that are truly positive) and recall (the proportion of true positives that are correctly identified). Jaccard index measures the degree of overlap between predicted and reference sets by dividing the size of their intersection by the size of their union; values closer to 1 indicate a higher concordance between the two.

### Human bladder cancer sample Visium HD data collection

Pre-treatment formalin-fixed paraffin-embedded (FFPE) tissue blocks were obtained from patients diagnosed with muscle-invasive bladder cancer (MIBC). All samples were collected under an approved IRB protocol (PRO00037670), and written informed consent was obtained from all participants prior to tissue collection. Standard-of-care procedures were used to preserve and process the tissues. For spatial transcriptomics, 10 *μm* sections were cut from the FFPE blocks and mounted onto Visium HD Spatial Gene Expression slides (10x Genomics). Sections were prepared in accordance with the manufacturer’s guidelines (10x Genomics). All subsequent steps were performed following the Visium HD sample preparation protocol.

## Data Availability

All the source codes are attached as supplementary files. We will release them on GitHub upon acceptance of the manuscript.
